# Expanding spectrum of illness due to community-associated methicillin-resistant *Staphylococcus aureus*: a case report

**DOI:** 10.4076/1757-1626-2-7437

**Published:** 2009-08-17

**Authors:** Russell Kempker, Lorenzo Difrancesco, Alejandro Martin-Gorgojo, Carlos Franco-Paredes

**Affiliations:** 1 Department of Infectious Diseases, Emory University School of Medicine550 Peachtree Street, MOT 7^th^ Floor Atlanta, GA 30308USA; 2 Department of General Medicine, Emory University School of Medicine 69 Jesse Hill Jr, Drive, S.E. AtlantaGA 30303USA; 3 University of Valencia Medical SchoolAvda Blasco Ibanez 15, Valencia 460101Spain

## Abstract

**Introduction:**

Xanthogranulomatous pyelonephritis is a rarely identified form of chronic pyelonephritis usually caused by gram-negative organisms. Herein, we report the first case caused by infection due to community-associated methicillin-resistant *Staphylococcus aureus* strain.

**Case presentation:**

An HIV-infected female with a history of intravenous drug use presented with a three month history of abdominal pain, distension, and fever. Computerized tomography of the abdomen revealed a large multicystic right kidney mass. During surgical exploration an intrarenal abscess was identified. Cultures revealed the presence of community-associated methicillin-resistant *Staphylococcus aureus*. Histopathological examination demonstrated xanthogranulomatous disease. The patient was successfully treated with nephrectomy and a prolonged antibiotic combination regimen with vancomycin and linezolid.

**Conclusion:**

Our case illustrates the escalating spectrum of illness caused by infection due to community-associated methicillin-resistant *Staphylococcus aureus*.

## Introduction

Infection with methicillin-resistant *Staphylococcus aureus* (MRSA) was first reported in the early 1960s, shortly after the introduction of methicillin and evolved into an important cause of nosocomial infection. Over the last three decades the incidence of MRSA infection in the United States and worldwide has dramatically increased [1]. The rise in MRSA infections has become an economic burden on the US health care system and is responsible for increasing amounts of patient morbidity and mortality. In a recent study by Klevens et al, it was estimated that 94,360 cases of invasive MRSA infection associated with death in 18,650 cases occurred in the United States in 2005 [2]. Reports have also shown MRSA infections to cause increased rates of morbidity and mortality as compared to methicillin sensitive *Staphylococcus aureus* infections [3,4]. More recently MRSA infection has begun to emerge in the community setting, with some cases occurring in patients who have never been hospitalized and who have no risk factors for MRSA infection. These cases have been defined as community-associated (CA) MRSA infections [5].

Strains of MRSA causing epidemiologically defined community associated and health care associated (HA) MRSA disease have been shown to be distinct. In a study comparing CA and HA MRSA infection it was shown that isolates from the community were more susceptible to non-B Lactam antibiotics, and more likely to possess *staphylococcal chromosomal cassette mec* IV and contain genes encoding for Panton-Valentine leukocidin (PVL) toxin [6]. Molecular typing studies with pulse field gel electrophoresis have defined one pulsed-field type that accounts for most CA MRSA disease, specified as type USA 300. In addition CA MRSA infections also tend to be found more often among healthy, community dwelling persons without traditional risk factors for MRSA [7].

The rapid evolution of CA MRSA infections has taken place over the last decade and a half. In 1993, MRSA infections among western Australian aborigines with no health care exposure were reported [8]. In 1999, four cases of rapidly fatal CA MRSA infections in children from the United States were described [9]. These reports heralded the rising epidemic of CA MRSA infections. In a recent population based surveillance study of three communities in 2001-2002 the CDC found between 8-20% of all MRSA infections were caused by CA MRSA and an annual disease incidence as high as 25.7/100,000 persons in the general population of Atlanta [10]. In analysis of all community onset skin and soft tissue infections presenting to a county hospital emergency room in Atlanta, King et al. found that CA MRSA caused approximately two-thirds of the cases [11]. While the majority of CA MRSA infections are skin and soft tissue infections, CAMRSA may also cause severe invasive disease [2,6]. Reports of CAMRSA causing life threatening infections include cases of necrotizing pneumonia and necrotizing fasciitis [12,13]. Other emerging clinical syndromes associated with CA MRSA include Waterhouse-Friderichsen syndrome, empyema, septic thrombophlebitis, prostatic abscess, and bacteremia [14]. In a large case series of CA MRSA infections, urinary tract infections have been reported to cause up to 4% of CA MRSA infections but none of these cases led to xanthogranulomatous pyelonephritis (XGP) [10]. This condition is typically caused by enteric gram-negative bacilli most commonly *Proteus mirabilis or Escherichia coli*. The pathogenesis of XGP is believed to involve a combination of nephrolithiasis, obstruction, and ascending infection [15]. We were therefore interested in reporting a case of XGP associated with CA MRSA in an HIV-infected patient.

## Case presentation

A 52-year-old African American HIV infected female presented with three months of progressive abdominal pain, distension, and fever. The patient had never been on antiretroviral therapy and had a CD4 T-cell count of 396 cells/μL at the time of admission with an unknown HIV RNA viral load. She had a history of discoid lupus, hepatitis C, active tobacco, alcohol, and intravenous drug use (IVDU). The patient had not been hospitalized or seen in outpatient clinics in the previous year, and was not taking any medications. She reported living with a family member and denied any sick contacts.

On examination, the patient was found to be febrile at 39.6°C. Physical examination was remarkable for a distended abdomen with right-sided costo-vertebral tenderness and right lower extremity track marks. On laboratory analysis, the leukocyte count was 9240/mcl with 71% segmented neutrophils, the hemoglobin 8.3 gm/dl, creatinine 1.2 mg/dl, and albumin 1.9 gm/dl. The rest of the chemistry panel including amylase and lipase were within normal. The urinanalysis revealed 5-10 RBC/hpf with no WBC’s, a negative HCG pregnancy test, and a drug screen positive for cocaine and opiates.

**Figure 1.**
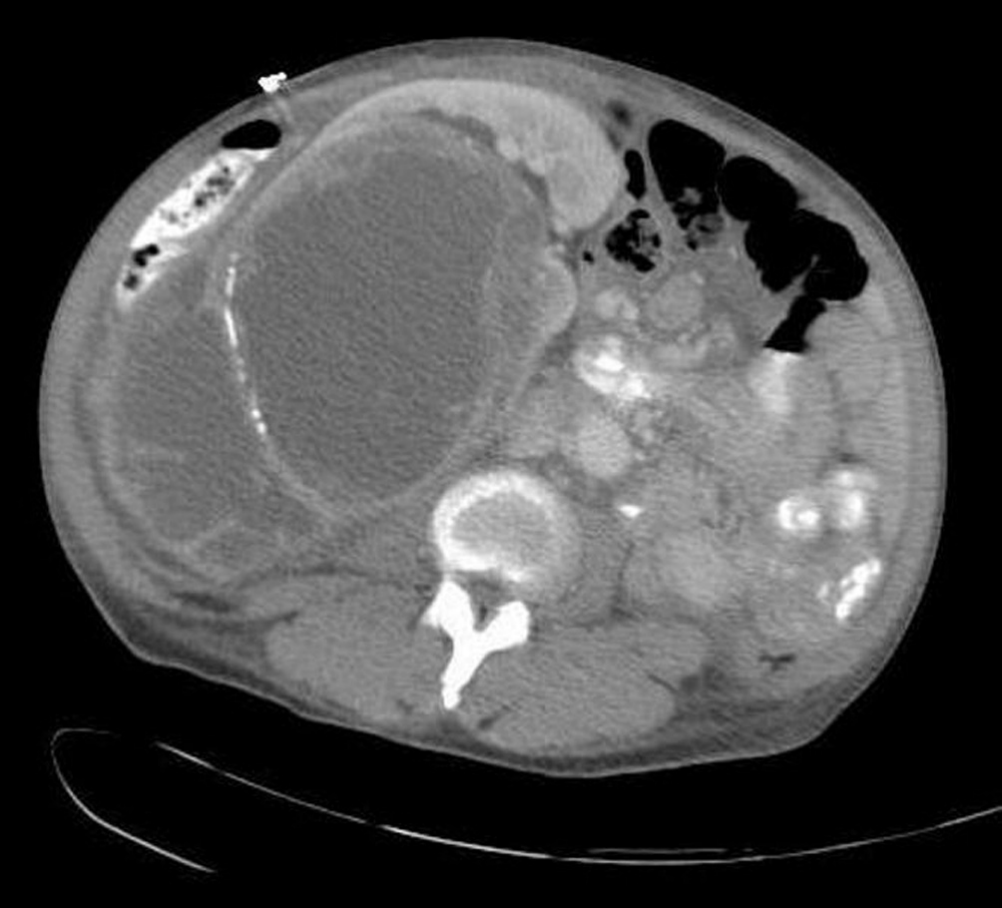
Abdominal CT scan revealing a large, complex cystic mass with multiple enhancing septations and septae calcifications arising from the right kidney.

**Figure 2.**
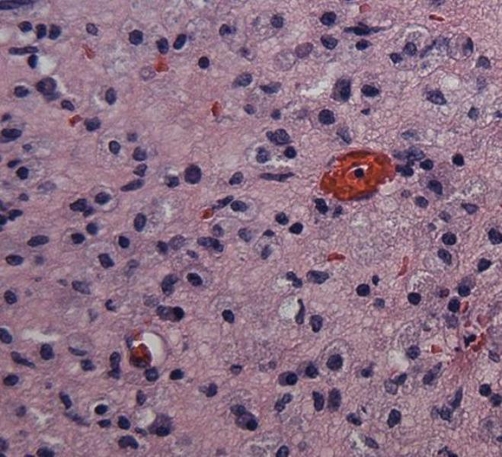
Histopathology at 40× magnification reveals characteristic foamy histiocytes as well as scattered plasma cells and lymphocytes and the absence of any normal renal tissue.

The patient was admitted to the hospital for further work up. A computed tomography (CT) scan of the abdomen and pelvis was done which revealed a 17.5 cm * 12.8 cm multicystic right-sided renal mass with septations and no evidence of invasion (Figure 1). The initial diagnostic impression was consistent with a multilocular cystic nephroma. The patient continued to be febrile and was started empirically on vancomycin and piperacllin-tazobactam. Urine and blood cultures sent on admission and multiple repeat blood cultures throughout the hospital stay came back as negative. Two weeks into her hospital stay the patient underwent surgical exploration. A large intrarenal abscess drained over three liters of purulent material and nephrectomy was subsequently performed. Cultures grew out MRSA sensitive to erythromycin, clindamycin, trimethoprim-sulfamethoxazole, levoquin, gentamycin, vancomycin, and linezolid. Based on this antibiogram we concluded that this bacterial isolate was consistent with a CA MRSA strain. Pathologic examination showed significant necrotic tissue, an abundance of inflammatory cells, and foamy histiocytes or xanthoma cells all characteristic of XGP (Figure 2). After surgery, the patient received a three-week course of vancomycin switched to linezolid upon discharge with complete recovery.

## Discussion

Xanthogranulomatous pyelonephritis was first described as a pathological entity in 1916. It is a rare form of chronic pyelonephritis that results in diffuse renal parenchymal obstruction. The disease process is traditionally associated with nephrolithiasis, obstruction, and infection with enteric *gram-negative bacilli*, typically *Proteus mirabilis* or *Escherichia coli.* It is unclear if the obstruction or the infection is the inciting pathological event [15]. *Staphylococcus aureus* has rarely been reported as a cause of XGP and to our knowledge there are no previous reports in the literature of CA MRSA causing XGP.

Our patient had evidence of XGP caused by MRSA. The patients MRSA infection met the CDC epidemiological criteria of CA MRSA, as she had no hospital exposure or medical procedures such as surgery, dialysis, or catheter placement during the last year [5]. CA MRSA has also been shown to be microbiology distinct from health care associated MRSA infection. In a study by Naimi et al, comparing the antimicrobial susceptibility pattern of CA MRSA and HA MRSA infections it was found that susceptibility to four non-beta Lactam antibiotics was able to predict a CA MRSA strain with an odds ratio of 2.44 (95% CI, 1.35-3.86) [6]. Our patient’s strain of MRSA met this microbiological criterion for CA MRSA.

In 1971, Povsil and colleagues were able to experimentally induce XGP in rats by ligating their ureters and injecting *Escherichia coli* into their tails [16]. Few experiments to further elucidate the mechanisms of pathogenesis have been done since. In prior case series of XGP, *Staphylococcus aureus* has only rarely been identified as a causative agent, and there has been only one case report of XGP due to MRSA specifically [15,17]. Our patient’s main risk factor for infection was her history of intravenous drug use, which has been associated with outbreaks of CA MRSA infection [18]. She presented with the common manifestations of fever and flank pain. We presume her CA MRSA infection derived from a skin source, given her history of IVDU and negative blood and urine cultures upon admission. It is also plausible that she had a prior episode of undetectable bacteremia and seeded her kidney at that time. Abreu et al, recently reported 13 cases of retroperitoneal infections causes by CA MRSA in which all cases had preceding skin infections, negative urine cultures, and only three of the thirteen had positive blood cultures [19]. These findings demonstrate the possibility that retroperitoneal CA MRSA infections can come from the skin.

Typical radiological findings of XGP include multiple fluid filled cavities in the affected kidney with extension of the disease into the perinephric space. As with our case, XGP is often confused with other disorders including renal tuberculosis, pyelonephritis with stones, and renal tumors and is commonly not included in the differential diagnosis [20]. XGP is a pathological entity and thus can only be definitively diagnosed by pathological examination. Our patient’s pathology exhibited the classic finding of lipid-laden macrophages mixed with chronic inflammatory signs. It is hypothesized that bacterial infection provokes tissue destruction and liberates pericalyceal and necrotic material, attracting inflammatory cells to the area, including macrophages, which take up and become filled with lipid debris [15]. Our patient made a complete recovery with the combination of open nephrectomy and a prolonged antibiotic course. Nephrectomy is considered the definitive and curative treatment for XGP with antibiotics serving as adjunctive therapy. There have been a few case reports of successful outcomes with antibiotics alone in focal forms of XGP [15]. In addition there have been reports of good outcomes and shorter hospital stays with laparoscopic nephrectomies compared to open nephrectomies [21].

In summary, the clinical spectrum of CA MRSA infections continues to expand. To our knowledge, this is the first report of a patient that developed XGP caused by CA MRSA infection. While the majority of cases produce skin and soft tissue infections there are increasing reports of new suppurative manifestations of CA MRSA infections. Physicians need to be aware of the variety of CA MRSA infections and keep CA MRSA in the differential when dealing with a potential case of XGP.

## Abbreviations

CA: community-associated
HA: healthcare-associated
IVDU: intravenous drug use
MRSA: methicillin-resistant *Staphylococcal aureus*
XGP: xanthogranulomatous pyelonephritis
